# *cis*-1,2-Bis{[4-(4-pyrid­yl)pyrimidin-2-yl]sulfanylmeth­yl}benzene

**DOI:** 10.1107/S1600536809045450

**Published:** 2009-11-04

**Authors:** Hua-Ze Dong, Hai-Bin Zhu, Shao-Hua Gou

**Affiliations:** a Deparment of Chemistry and Chemical Engineering, Hefei Normal University, Hefei 230061, People’s Republic of China; b School of Chemistry and Chemical Engineering, Southeast University, Nanjing 211189, People’s Republic of China

## Abstract

The mol­ecular skeleton of the title mol­ecule, C_26_H_20_N_6_S_2_, adopts a *cis* conformation with the two arms positioned on one side of the benzene ring plane. Intra­molecular π–π inter­actions between the pyrimidine rings [centroid–centroid distance = 3.654 (2) Å] and between the pyridine rings [centroid–centroid distance = 3.775 (2) Å] help to set the mol­ecular conformation; the pyrimidine rings, as well as the pyridine rings, are nearly parallel, forming dihedral angles of 4.12 (14) and 2.46 (14)°, respectively.

## Related literature

For related compounds, see: Dong *et al.* (2008 ▶, 2009 ▶); Huang *et al.* (2007 ▶).
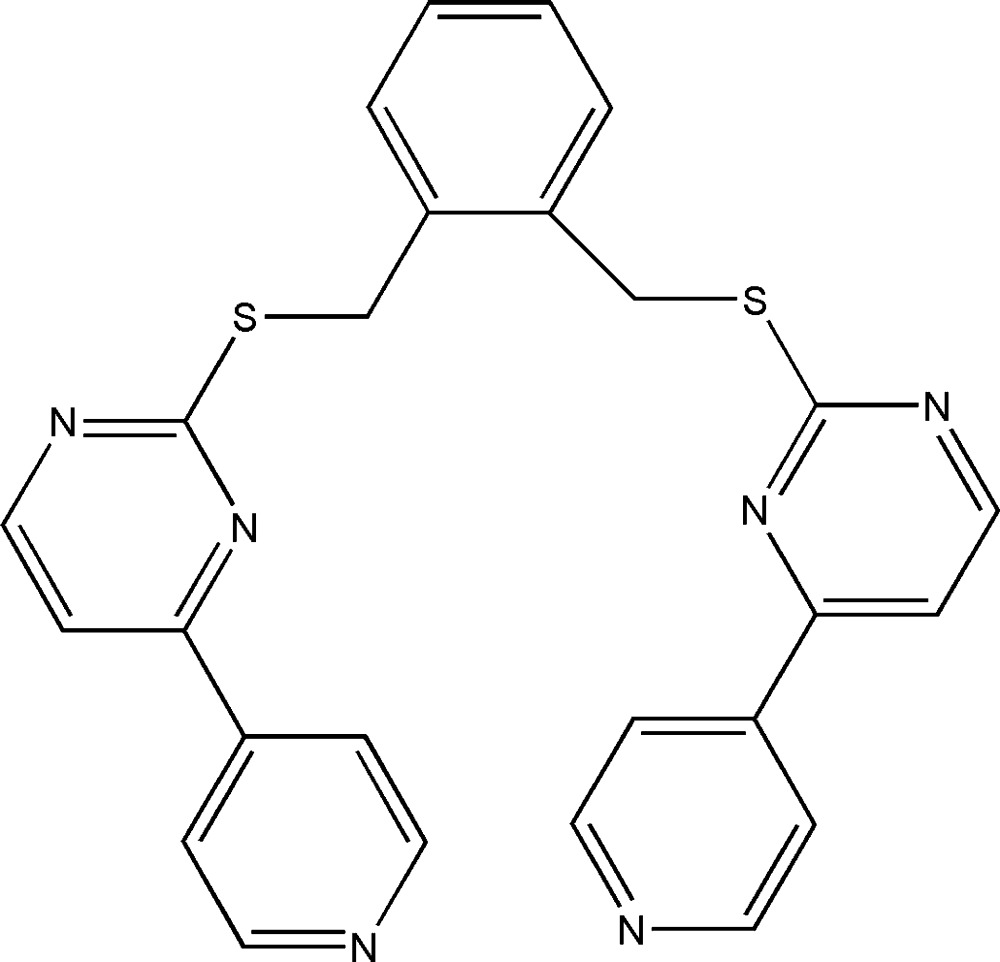


## Experimental

### 

#### Crystal data

C_26_H_20_N_6_S_2_
*M* *_r_* = 480.62
Monoclinic, 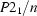

*a* = 8.6078 (11) Å
*b* = 27.102 (3) Å
*c* = 10.0282 (12) Å
β = 103.661 (2)°
*V* = 2273.3 (5) Å^3^
*Z* = 4
Mo *K*α radiation
μ = 0.26 mm^−1^
*T* = 291 K
0.32 × 0.18 × 0.16 mm

#### Data collection

Bruker SMART CCD area-detector diffractometer
Absorption correction: multi-scan (*SADABS*; Bruker, 2000 ▶) *T*_min_ = 0.917, *T*_max_ = 0.966
13064 measured reflections
4903 independent reflections
3067 reflections with *I* > 2σ(*I*)
*R*_int_ = 0.052

#### Refinement

*R*[*F* ^2^ > 2σ(*F* ^2^)] = 0.052
*wR*(*F* ^2^) = 0.108
*S* = 0.90
4903 reflections
307 parameters
H-atom parameters constrained
Δρ_max_ = 0.32 e Å^−3^
Δρ_min_ = −0.22 e Å^−3^

### 

Data collection: *SMART* (Bruker, 2000 ▶); cell refinement: *SAINT* (Bruker, 2000 ▶); data reduction: *SAINT*; program(s) used to solve structure: *SHELXTL* (Sheldrick, 2008 ▶); program(s) used to refine structure: *SHELXTL*; molecular graphics: *SHELXTL*; software used to prepare material for publication: *SHELXTL*.

## Supplementary Material

Crystal structure: contains datablocks I, global. DOI: 10.1107/S1600536809045450/cv2646sup1.cif

Structure factors: contains datablocks I. DOI: 10.1107/S1600536809045450/cv2646Isup2.hkl

Additional supplementary materials:  crystallographic information; 3D view; checkCIF report

## Figures and Tables

**Fig. 1.**
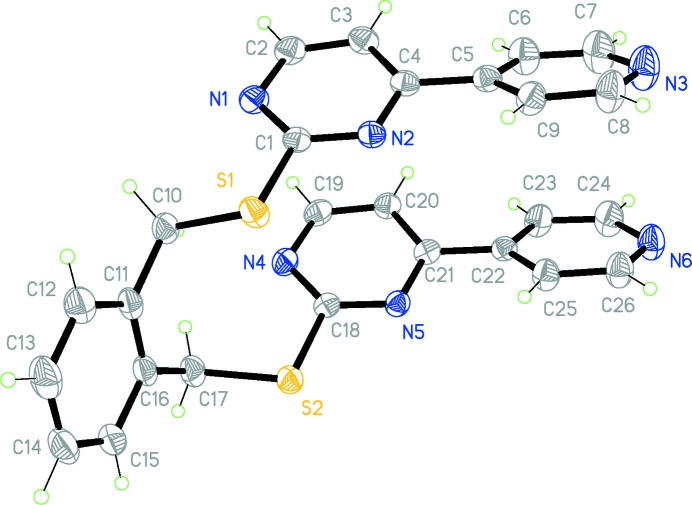
The molecular structure of the title compound showing 30% probability displacement ellipsoids and the atom-numbering scheme.

## supplementary crystallographic information

### Comment

Remarkable attention has been paid to the rational design and assembly of new coordination polymers with heterocyclic thiolates or thioethers in recent years. In our previous work, we reported aserials dithioether ligands (Dong *et al.*, 2008; 2009; Huang *et al.*, 2007). As our continuing study, herein we report the molecular structure of the title compound - the newly synthesized ligand derived from 4-(4-pyridinyl)pyrimidine-2-thiol.

The molecular structure of the title compound is shown in Fig. 1. The molecule adopts a *cis* conformation with two arms positioned on one side of the benzene ring plane. It is noted that intramolecular π-π interactions between the pyrimidinyl rings [centroid-centroid distance of 3.654 (2) Å] and between the pyridinyl rings [centroid-centroid distance of 3.775 (2) Å] set the molecular conformation - the pyrimidinyl rings, as well as the pyridinyl ones, are nearly parallel forming dihedral angles of 4.12 (14)° and 2.46 (14)°, respectively.

### Experimental

All solvents and chemicals were of analytical grade and were used without further purification. The title compound was prepared by similar procedure reported in the literature (Dong *et al.*, 2008; 2009), To a solution of 4-(4-pyridinyl)pyrimidine-2-thiol (3.78 g, 20 mmol) and sodium hydroxide (0.80 g, 20 mmol) in dry ethanol (300 ml), 1,2-bis(bromomethyl)benzene (2.64 g, 10 mmol) in CCl_4_ (30 ml) was added. The mixture was stirred and refluxed for 8 h. After cooling, precipitates were filtered, washed in water and ethanol, and dried in vacuum. Anal. Calcd for C_26_H_20_N_6_S_2_: C, 64.98; H, 4.19; N, 17.49%. Found: C, 65.07; H, 4.52; N, 17.34%. Single crystals of ligang suitable for X-ray diffraction were grown from methanol solution by slow evaporation in air at room temperature.

### Refinement

All hydrogen atoms were geometrically positioned (C—H 0.93–0.97 Å) and refined as riding, with *U*_iso_(H)=1.2*U*_eq_(C).

### Crystal data

**Table:**

C_26_H_20_N_6_S_2_	*F*(000) = 1000.0
*M**_r_* = 480.62	*D*_x_ = 1.404 Mg m^−^^3^
Monoclinic, *P*2_1_/*n*	Mo *K*α radiation, λ = 0.71073 Å
Hall symbol: -P 2yn	Cell parameters from 4903 reflections
*a* = 8.6078 (11) Å	θ = 2.2–27.0°
*b* = 27.102 (3) Å	µ = 0.26 mm^−^^1^
*c* = 10.0282 (12) Å	*T* = 291 K
β = 103.661 (2)°	Block, pale yellow
*V* = 2273.3 (5) Å^3^	0.32 × 0.18 × 0.16 mm
*Z* = 4	

### Data collection

**Table:**

Bruker SMART CCD area-detector diffractometer	4903 independent reflections
Radiation source: fine-focus sealed tube	3067 reflections with *I* > 2σ(*I*)
graphite	*R*_int_ = 0.052
φ and ω scans	θ_max_ = 27.0°, θ_min_ = 2.2°
Absorption correction: multi-scan (*SADABS*; Bruker, 2000)	*h* = −10→8
*T*_min_ = 0.917, *T*_max_ = 0.966	*k* = −34→31
13064 measured reflections	*l* = −12→12

### Refinement

**Table:**

Refinement on *F*^2^	Primary atom site location: structure-invariant direct methods
Least-squares matrix: full	Secondary atom site location: difference Fourier map
*R*[*F*^2^ > 2σ(*F*^2^)] = 0.052	Hydrogen site location: inferred from neighbouring sites
*wR*(*F*^2^) = 0.108	H-atom parameters constrained
*S* = 0.90	*w* = 1/[σ^2^(*F*_o_^2^) + (0.0421*P*)^2^] where *P* = (*F*_o_^2^ + 2*F*_c_^2^)/3
4903 reflections	(Δ/σ)_max_ = 0.001
307 parameters	Δρ_max_ = 0.32 e Å^−^^3^
0 restraints	Δρ_min_ = −0.22 e Å^−^^3^

### Special details

**Table:**

Geometry. All e.s.d.'s (except the e.s.d. in the dihedral angle between two l.s. planes) are estimated using the full covariance matrix. The cell e.s.d.'s are taken into account individually in the estimation of e.s.d.'s in distances, angles and torsion angles; correlations between e.s.d.'s in cell parameters are only used when they are defined by crystal symmetry. An approximate (isotropic) treatment of cell e.s.d.'s is used for estimating e.s.d.'s involving l.s. planes.
Refinement. Refinement of *F*^2^ against ALL reflections. The weighted *R*-factor *wR* and goodness of fit *S* are based on *F*^2^, conventional *R*-factors *R* are based on *F*, with *F* set to zero for negative *F*^2^. The threshold expression of *F*^2^ > σ(*F*^2^) is used only for calculating *R*-factors(gt) *etc*. and is not relevant to the choice of reflections for refinement. *R*-factors based on *F*^2^ are statistically about twice as large as those based on *F*, and *R*- factors based on ALL data will be even larger.

### Fractional atomic coordinates and isotropic or equivalent isotropic displacement parameters (Å^2^)

**Table:**

	*x*	*y*	*z*	*U*_iso_*/*U*_eq_	
C1	0.7345 (3)	0.22381 (8)	0.8229 (2)	0.0384 (5)	
C2	0.9748 (3)	0.25202 (9)	0.9324 (2)	0.0494 (6)	
H2	1.0799	0.2469	0.9807	0.059*	
C3	0.9230 (3)	0.29935 (8)	0.9059 (2)	0.0474 (6)	
H3	0.9909	0.3259	0.9351	0.057*	
C4	0.7668 (3)	0.30680 (8)	0.8343 (2)	0.0411 (6)	
C5	0.6967 (3)	0.35654 (8)	0.8048 (2)	0.0436 (6)	
C6	0.7521 (3)	0.39620 (9)	0.8867 (3)	0.0623 (8)	
H6	0.8355	0.3922	0.9640	0.075*	
C7	0.6845 (4)	0.44177 (10)	0.8545 (3)	0.0774 (9)	
H7	0.7240	0.4680	0.9125	0.093*	
C8	0.5126 (3)	0.41215 (9)	0.6685 (3)	0.0690 (8)	
H8	0.4291	0.4172	0.5919	0.083*	
C9	0.5716 (3)	0.36519 (8)	0.6935 (3)	0.0529 (7)	
H9	0.5275	0.3394	0.6357	0.063*	
C10	0.6875 (3)	0.12421 (7)	0.8693 (2)	0.0472 (6)	
H10A	0.7817	0.1124	0.8418	0.057*	
H10B	0.7188	0.1342	0.9648	0.057*	
C11	0.5630 (3)	0.08400 (8)	0.8510 (2)	0.0443 (6)	
C12	0.5488 (3)	0.05264 (9)	0.7392 (3)	0.0592 (7)	
H12	0.6183	0.0560	0.6815	0.071*	
C13	0.4334 (4)	0.01686 (9)	0.7133 (3)	0.0687 (8)	
H13	0.4248	−0.0038	0.6379	0.082*	
C14	0.3317 (3)	0.01133 (9)	0.7966 (3)	0.0660 (8)	
H14	0.2519	−0.0126	0.7777	0.079*	
C15	0.3473 (3)	0.04123 (8)	0.9091 (3)	0.0566 (7)	
H15	0.2794	0.0367	0.9677	0.068*	
C16	0.4617 (3)	0.07813 (8)	0.9376 (2)	0.0446 (6)	
C17	0.4698 (3)	0.11019 (7)	1.0609 (2)	0.0502 (6)	
H17A	0.5805	0.1184	1.1017	0.060*	
H17B	0.4283	0.0921	1.1285	0.060*	
C18	0.4950 (3)	0.21357 (8)	1.0767 (2)	0.0380 (5)	
C19	0.7376 (3)	0.24131 (9)	1.1805 (2)	0.0484 (6)	
H19	0.8442	0.2358	1.2238	0.058*	
C20	0.6847 (3)	0.28872 (8)	1.1637 (2)	0.0446 (6)	
H20	0.7523	0.3151	1.1955	0.053*	
C21	0.5267 (3)	0.29608 (8)	1.0977 (2)	0.0393 (5)	
C22	0.4562 (3)	0.34572 (8)	1.0709 (2)	0.0399 (5)	
C23	0.5154 (3)	0.38590 (9)	1.1509 (3)	0.0589 (7)	
H23	0.6024	0.3823	1.2253	0.071*	
C24	0.4452 (4)	0.43133 (9)	1.1201 (3)	0.0701 (9)	
H24	0.4868	0.4579	1.1761	0.084*	
C25	0.3284 (3)	0.35403 (8)	0.9626 (2)	0.0497 (6)	
H25	0.2835	0.3282	0.9052	0.060*	
C26	0.2679 (3)	0.40072 (9)	0.9402 (3)	0.0597 (7)	
H26	0.1816	0.4054	0.8656	0.072*	
N1	0.8822 (2)	0.21295 (7)	0.89261 (18)	0.0458 (5)	
N2	0.6713 (2)	0.26820 (6)	0.79073 (17)	0.0405 (5)	
N3	0.5667 (3)	0.45081 (8)	0.7465 (3)	0.0771 (8)	
N4	0.6443 (2)	0.20240 (7)	1.13813 (19)	0.0463 (5)	
N5	0.4294 (2)	0.25802 (6)	1.05275 (17)	0.0394 (5)	
N6	0.3221 (3)	0.43947 (7)	1.0157 (2)	0.0640 (6)	
S1	0.60063 (8)	0.17575 (2)	0.76342 (6)	0.04810 (19)	
S2	0.35623 (8)	0.16649 (2)	1.01669 (6)	0.04634 (19)	

### Atomic displacement parameters (Å^2^)

**Table:**

	*U*^11^	*U*^22^	*U*^33^	*U*^12^	*U*^13^	*U*^23^
C1	0.0353 (14)	0.0411 (13)	0.0382 (13)	−0.0043 (11)	0.0074 (11)	0.0000 (10)
C2	0.0363 (16)	0.0586 (16)	0.0487 (15)	−0.0038 (12)	0.0005 (12)	0.0005 (12)
C3	0.0409 (16)	0.0496 (15)	0.0480 (15)	−0.0112 (12)	0.0031 (12)	−0.0033 (11)
C4	0.0424 (15)	0.0443 (13)	0.0377 (13)	−0.0058 (12)	0.0116 (11)	−0.0023 (10)
C5	0.0423 (16)	0.0436 (14)	0.0462 (14)	−0.0055 (11)	0.0131 (12)	−0.0066 (11)
C6	0.068 (2)	0.0518 (16)	0.0583 (17)	−0.0030 (15)	−0.0019 (14)	−0.0126 (13)
C7	0.090 (3)	0.0455 (17)	0.086 (2)	−0.0035 (16)	−0.001 (2)	−0.0243 (15)
C8	0.063 (2)	0.0504 (16)	0.081 (2)	0.0088 (14)	−0.0070 (16)	−0.0122 (15)
C9	0.0461 (17)	0.0422 (14)	0.0656 (17)	−0.0020 (12)	0.0036 (14)	−0.0122 (12)
C10	0.0394 (15)	0.0401 (13)	0.0559 (15)	0.0036 (11)	−0.0010 (12)	0.0066 (11)
C11	0.0447 (16)	0.0305 (12)	0.0509 (15)	0.0019 (11)	−0.0025 (12)	0.0012 (11)
C12	0.064 (2)	0.0480 (15)	0.0640 (18)	−0.0002 (14)	0.0131 (15)	−0.0030 (13)
C13	0.079 (2)	0.0455 (16)	0.072 (2)	−0.0027 (15)	−0.0014 (18)	−0.0151 (14)
C14	0.058 (2)	0.0367 (15)	0.094 (2)	−0.0075 (13)	−0.0005 (17)	−0.0006 (15)
C15	0.0512 (18)	0.0380 (14)	0.079 (2)	0.0012 (12)	0.0129 (15)	0.0064 (13)
C16	0.0457 (16)	0.0304 (12)	0.0526 (15)	0.0027 (11)	0.0016 (12)	0.0056 (11)
C17	0.0563 (17)	0.0387 (13)	0.0527 (16)	0.0057 (12)	0.0071 (13)	0.0101 (11)
C18	0.0354 (15)	0.0429 (13)	0.0355 (13)	0.0000 (11)	0.0084 (11)	−0.0030 (10)
C19	0.0335 (15)	0.0601 (16)	0.0471 (15)	0.0018 (13)	0.0005 (12)	0.0007 (12)
C20	0.0357 (15)	0.0464 (14)	0.0473 (14)	−0.0037 (11)	0.0014 (12)	−0.0047 (11)
C21	0.0379 (14)	0.0453 (13)	0.0340 (13)	−0.0012 (11)	0.0070 (11)	−0.0043 (10)
C22	0.0364 (14)	0.0410 (13)	0.0417 (13)	−0.0033 (11)	0.0080 (11)	−0.0054 (10)
C23	0.0475 (17)	0.0524 (16)	0.0654 (18)	0.0002 (13)	−0.0096 (14)	−0.0141 (13)
C24	0.065 (2)	0.0455 (16)	0.088 (2)	−0.0040 (14)	−0.0062 (17)	−0.0218 (14)
C25	0.0514 (17)	0.0407 (14)	0.0511 (15)	0.0003 (12)	0.0003 (13)	−0.0089 (11)
C26	0.0584 (19)	0.0521 (16)	0.0590 (17)	0.0073 (14)	−0.0057 (14)	−0.0017 (13)
N1	0.0354 (13)	0.0505 (12)	0.0475 (12)	−0.0011 (10)	0.0016 (10)	0.0037 (9)
N2	0.0357 (12)	0.0397 (11)	0.0440 (11)	−0.0041 (9)	0.0054 (9)	−0.0016 (9)
N3	0.079 (2)	0.0508 (14)	0.0889 (19)	0.0089 (13)	−0.0057 (15)	−0.0148 (13)
N4	0.0392 (13)	0.0472 (11)	0.0494 (12)	0.0050 (10)	0.0041 (10)	0.0038 (9)
N5	0.0350 (12)	0.0394 (11)	0.0420 (11)	0.0016 (9)	0.0053 (9)	−0.0027 (8)
N6	0.0645 (17)	0.0434 (13)	0.0754 (16)	0.0052 (11)	−0.0007 (13)	−0.0055 (11)
S1	0.0409 (4)	0.0417 (3)	0.0538 (4)	−0.0057 (3)	−0.0044 (3)	0.0051 (3)
S2	0.0425 (4)	0.0402 (3)	0.0539 (4)	−0.0003 (3)	0.0067 (3)	−0.0024 (3)

### Geometric parameters (Å, °)

**Table:**

C1—N2	1.328 (2)	C13—H13	0.9300
C1—N1	1.331 (3)	C14—C15	1.369 (3)
C1—S1	1.748 (2)	C14—H14	0.9300
C2—N1	1.329 (3)	C15—C16	1.385 (3)
C2—C3	1.364 (3)	C15—H15	0.9300
C2—H2	0.9300	C16—C17	1.500 (3)
C3—C4	1.381 (3)	C17—S2	1.810 (2)
C3—H3	0.9300	C17—H17A	0.9700
C4—N2	1.338 (2)	C17—H17B	0.9700
C4—C5	1.478 (3)	C18—N4	1.323 (3)
C5—C6	1.369 (3)	C18—N5	1.328 (2)
C5—C9	1.375 (3)	C18—S2	1.754 (2)
C6—C7	1.371 (3)	C19—N4	1.333 (3)
C6—H6	0.9300	C19—C20	1.360 (3)
C7—N3	1.320 (3)	C19—H19	0.9300
C7—H7	0.9300	C20—C21	1.379 (3)
C8—N3	1.324 (3)	C20—H20	0.9300
C8—C9	1.372 (3)	C21—N5	1.339 (2)
C8—H8	0.9300	C21—C22	1.474 (3)
C9—H9	0.9300	C22—C25	1.369 (3)
C10—C11	1.509 (3)	C22—C23	1.377 (3)
C10—S1	1.807 (2)	C23—C24	1.374 (3)
C10—H10A	0.9700	C23—H23	0.9300
C10—H10B	0.9700	C24—N6	1.321 (3)
C11—C16	1.377 (3)	C24—H24	0.9300
C11—C12	1.389 (3)	C25—C26	1.367 (3)
C12—C13	1.368 (3)	C25—H25	0.9300
C12—H12	0.9300	C26—N6	1.314 (3)
C13—C14	1.354 (4)	C26—H26	0.9300
			
N2—C1—N1	127.8 (2)	C14—C15—H15	119.2
N2—C1—S1	113.12 (17)	C16—C15—H15	119.2
N1—C1—S1	119.04 (17)	C11—C16—C15	118.6 (2)
N1—C2—C3	123.0 (2)	C11—C16—C17	122.7 (2)
N1—C2—H2	118.5	C15—C16—C17	118.7 (2)
C3—C2—H2	118.5	C16—C17—S2	111.68 (15)
C2—C3—C4	118.2 (2)	C16—C17—H17A	109.3
C2—C3—H3	120.9	S2—C17—H17A	109.3
C4—C3—H3	120.9	C16—C17—H17B	109.3
N2—C4—C3	120.2 (2)	S2—C17—H17B	109.3
N2—C4—C5	117.2 (2)	H17A—C17—H17B	107.9
C3—C4—C5	122.6 (2)	N4—C18—N5	128.1 (2)
C6—C5—C9	116.6 (2)	N4—C18—S2	120.10 (17)
C6—C5—C4	121.9 (2)	N5—C18—S2	111.83 (17)
C9—C5—C4	121.5 (2)	N4—C19—C20	123.3 (2)
C5—C6—C7	119.8 (2)	N4—C19—H19	118.4
C5—C6—H6	120.1	C20—C19—H19	118.4
C7—C6—H6	120.1	C19—C20—C21	117.4 (2)
N3—C7—C6	124.2 (2)	C19—C20—H20	121.3
N3—C7—H7	117.9	C21—C20—H20	121.3
C6—C7—H7	117.9	N5—C21—C20	121.2 (2)
N3—C8—C9	124.2 (2)	N5—C21—C22	116.3 (2)
N3—C8—H8	117.9	C20—C21—C22	122.4 (2)
C9—C8—H8	117.9	C25—C22—C23	116.7 (2)
C8—C9—C5	119.5 (2)	C25—C22—C21	120.9 (2)
C8—C9—H9	120.3	C23—C22—C21	122.4 (2)
C5—C9—H9	120.3	C24—C23—C22	119.7 (2)
C11—C10—S1	107.66 (15)	C24—C23—H23	120.1
C11—C10—H10A	110.2	C22—C23—H23	120.1
S1—C10—H10A	110.2	N6—C24—C23	123.7 (2)
C11—C10—H10B	110.2	N6—C24—H24	118.1
S1—C10—H10B	110.2	C23—C24—H24	118.1
H10A—C10—H10B	108.5	C26—C25—C22	119.1 (2)
C16—C11—C12	119.3 (2)	C26—C25—H25	120.4
C16—C11—C10	122.9 (2)	C22—C25—H25	120.4
C12—C11—C10	117.7 (2)	N6—C26—C25	125.1 (2)
C13—C12—C11	120.6 (3)	N6—C26—H26	117.4
C13—C12—H12	119.7	C25—C26—H26	117.4
C11—C12—H12	119.7	C2—N1—C1	114.35 (19)
C14—C13—C12	120.5 (3)	C1—N2—C4	116.4 (2)
C14—C13—H13	119.8	C7—N3—C8	115.6 (2)
C12—C13—H13	119.8	C18—N4—C19	114.42 (19)
C13—C14—C15	119.4 (3)	C18—N5—C21	115.62 (19)
C13—C14—H14	120.3	C26—N6—C24	115.6 (2)
C15—C14—H14	120.3	C1—S1—C10	103.16 (11)
C14—C15—C16	121.6 (3)	C18—S2—C17	104.16 (11)
